# Development of the Intervention Materials for the HomeStyles Obesity Prevention Program for Parents of Preschoolers

**DOI:** 10.3390/nu7085301

**Published:** 2015-08-10

**Authors:** Jennifer Martin-Biggers, Kim Spaccarotella, Colleen Delaney, Mallory Koenings, Gayle Alleman, Nobuko Hongu, John Worobey, Carol Byrd-Bredbenner

**Affiliations:** 1 Department of Nutritional Sciences, Rutgers University, 26 Nichol Avenue New Brunswick, NJ 08901, USA; E-Mails: jmartin@njaes.rutgers.edu (J.M.-B.); colleen.delaney@rutgers.edu (C.D.); koenings@aesop.rutgers.edu (M.K.); worobey@rci.rutgers.edu (J.W.); 2 Department of Biological Sciences, Kean University, 1000 Morris Avenue Union, NJ 07082, USA; E-Mail: kspaccar@kean.edu; 3 Department of Nutritional Sciences, University of Arizona, 406 Shantz Building, 1177 E. 4th Street, Tucson, AZ 85721-0038, USA; E-Mails: galleman@email.arizona.edu (G.A.); hongu@email.arizona.edu (N.H.)

**Keywords:** obesity prevention, preschool parents, home environment

## Abstract

Home environment is key to the development of obesity-preventing behaviors during childhood, yet few resources help preschool parents address factors at home associated with obesity risk. This paper describes creation of materials for an in-home intervention (HomeStyles) with this population. An advisory group of stakeholders and target audience members determined salient factors affecting childhood obesity to address in-home and developed program materials. The Social Cognitive Theory, Faith’s Core Behavior Change Strategies to Treat Childhood Obesity, Adult Learning Theory and motivational interviewing techniques guided development of 12 guides targeting strategies parents can use to shape the home environment. Interviews were conducted to determine effectiveness of the guides. Cognitive testing of guide design (*n* = 251) and content (*n* = 261) occurred in English and Spanish in New Jersey and Arizona with parents and home visitation staff who would present the guides. Interviews investigated perceptions of content usefulness and parent comprehension. Findings were also examined in light of theoretical underpinnings. Both home visitation staff and parents felt the guides were very readable and useful. Parents appreciated use of motivational interviewing techniques and Adult Learning Theory. Current research is testing these guides through an in-home, randomized control trial.

## 1. Introduction

The home environment plays a dominant role in the development of childhood lifestyle patterns and these patterns track across the growing years into adulthood [1,2,3,4,5]. As role models and family food gatekeepers, parents strongly influence food intake and physical activity behaviors of children [6,7,8,9,10,11,12,13,14] and are key influencers of obesity-preventing behaviors of children during the growing years [1,2,3,7,8,10,11,12,15,16,17,18,19,20,21,22,23,24,25,26,27,28,29,30,31,32,33,34,35,36,37,38,39,40,41,42,43,44,45,46]. For example, the influence of parental modeling of physical activity, beliefs about physical activity, and encouragement provided are important predictors of children’s physical activity levels [7,8]. In addition, children’s intakes of and preferences for vegetables, fruits, and calcium-rich foods are positively associated with availability at home, parental intake, and allowing the child to decide how much of the food to eat [16,17,18,19,20,21,22,23,24,25]. If household food supplies do not support healthful eating, it is unlikely families will be able to manage their weight [35]. Family meals appear to be particularly important in helping children develop healthy eating patterns [26,27,28,29,30,31,32,33,34].

Because Body Mass Index (BMI), as well as behaviors that directly affect BMI (*i.e.*, diet and exercise), track across childhood into adulthood, it is important for parents to actively safeguard children’s health by establishing positive obesity-preventive lifestyle habits [1,2,3,37,38,39,40,41,42,43]. According to the institute of Medicine (IOM), “families play a central role in childhood obesity prevention. Innovative approaches are needed to provide families with relevant obesity prevention information, particularly information that is practical, that is easily implemented, and that does not judge or lecture parents” ([15] p. 343). Raising awareness of the risks associated with obesity and providing realistic strategies parents can implement for preventing obesity are needed.

The IOM also recommends using an integrated multifactorial approach [15]. “Single-strategy obesity-prevention initiatives have had limited success, programs that target multiple behaviors may help reduce body weight and body fat among young children” [47]. For instance, preschoolers who regularly ate family meals, got adequate sleep, and limited screen-time had ~40% less risk of obesity than those exposed to none of these [48]. Practices that parents of preschoolers can shape within the home environment are related to diet (e.g., food availability, nutrient: energy density of available foods, feeding/mealtime patterns, response to children’s requests for advertised foods), physical activity (e.g., screen-time, age-appropriate exercise options, family activity patterns), and children’s sleep habits [7,8,26,27,28,29,30,31,32,33,34,49,50,51,52,53,54,55,56,57,58,59,60,61,62,63,64,65,66,67,68,69,70,71,72]. Closely related is childcare [73]. Young children in non-parental childcare settings have a greater risk of obesity, indicating parents may need opportunities to develop skills to advocate for provision of healthy foods and sufficient physical activity and naptime while children are in childcare [74,75,76].

To generate the most successful results, obesity prevention programs need to be placed within a family context and address multiple home environment lifestyle practices associated with weight status [77,78,79,80,81]. However, parents need more opportunities to gain relevant, practical, non-judgmental obesity prevention information that is easily implemented in their homes and hectic lifestyles [15]. Thus, HomeStyles, an in-home program that enables and motivates parents to shape their home environment and lifestyle behavioral practices (diet, exercise, sleep) to prevent excessive weight gain in their preschool children, was created. The purpose of this paper is to describe the process implemented in the creation of the intervention materials for the HomeStyles program.

## 2. Development of Intervention Materials

The approach taken in the development of this program was designed to maximize the likelihood that it would include the critical components of effective health interventions [13]. Namely, the approach incorporated participatory planning and implementation strategies (*i.e.*, advisory group, Adult Learning Theory, community-based participatory research principles), had a health behavior change theoretical basis (*i.e.*, Social Cognitive Theory), involved families with preschool children at every stage of development, and conveyed clear, actionable messages to participants using motivational interviewing principles.

### 2.1. Advisory Group

An advisory group was established to provide expert input during the design of program materials and to ensure all aspects of the program were congruent with current dietary guidance (*i.e.*, Dietary Guidelines for Americans and Healthy People 2020 recommendations) and best practices for communicating health messages [15,36,82,83,84,85,86]. The group included experts in child nutrition, nutrition education and communication, obesity prevention, child development/developmental psychology, pediatrics, early childhood education, exercise science, community-based participatory nutrition research, instructional media design, racially and culturally diverse audiences, outreach experts (e.g., *Parents as Teachers* and *Healthy Families* home visitors, Extension educators, Supplemental Nutrition Assistance Program education personnel), and members of the target audience (*i.e.*, parents of preschool children). Active participation of stakeholders, including gatekeepers (e.g., outreach personnel) and the target audience, during the development of health promotion programs is an important aspect of community-based participatory research principles and increases the likelihood of developing an acceptable, meaningful, sustainable program [87,88,89], yet stakeholder input is frequently missing in obesity prevention programs [15,78]. The advisory group regularly met with the HomeStyles research team in groups and individually using both in-person and electronic means.

### 2.2. Theoretical Underpinnings

Social Cognitive Theory, a health behavior change theory, and Faith’s Core Behavior Change Strategies to Treat Childhood Obesity were used to guide the development, characterization, refinement, and delivery of HomeStyles program materials [90,91,92]. Obesity preventive behaviors are practiced within the environment, which can support or subvert efforts [5,93,94,95,96,97]. Indeed, reciprocal determinism, an emphasis of Social Cognitive Theory, posits that a person’s characteristics, behavior, and the environment within which the behavior is performed simultaneously influence each other and that people have the ability to construct or modify environments to match their goals and desires [98]. The strong link between Social Cognitive Theory key concepts (e.g., self-efficacy, outcome expectancies, self-regulation, social norms, observation and modeling by important figures such as parents) and knowledge (as an antecedent) as well as application (*i.e.*, both intended and actual behavior within the environment) made this a suitable framework for an obesity prevention intervention based in the home [24,77,98,99,100,101,102,103]. The strength of research supporting the positive associations of self-efficacy, outcome expectancies, and self-regulatory behaviors with the practice of health-protective behaviors further supports this theory’s usefulness in creating HomeStyles program materials [98,99].

Motivational interviewing is an instructional strategy that uses non-judgmental, non-confrontational interviewing and empathetic, supportive, and reflective listening to help clients clarify strengths and valued goals and aspirations (e.g., raising healthy children). This information along with the client’s readiness to change enables the instructor to help clients imagine a better future, provide persuasive client-centered teaching strategies that evoke motivation to change, and help clients formulate plans to change that are consistent with the clients’ values [104,105,106,107,108]. Motivational interviewing is supported by over 100 randomized controlled trials across a range of populations and behaviors, including diet, exercise, and parenting [109,110,111,112,113,114,115]. And, the American Academy of Pediatrics’ *Prevention*, *Assessment*, *and Treatment of Child Overweight and Obesity Expert Committee* recommends using motivational interviewing [116]. Because this strategy also can be successfully adapted for written, self-instructional materials [117], it was used in the development of HomeStyles.

Adult Learning Theory also informed the presentation style used in HomeStyles [118,119,120]. Adult Learning Theory focuses on recognizing the characteristics of adult learners (parents) and creating learning experiences that motivate them to achieve personal goals. Adult learners are autonomous, self-directed, goal-oriented, relevancy-oriented, and practicality-oriented and bring life experiences and knowledge to the learning situation [121,122,123]. Effective instruction facilitates and motivates learning by actively involving adult learners as equal partners in the learning process, respecting their wealth of related knowledge and experiences, explaining why learning is important, seeking their perspectives about what is important (relevant, applicable, practical, useful) to learn, and encouraging them to focus on applying what they learn to solve problems and reach their goals.

### 2.3. Philosophical Basis

The philosophical basis for HomeStyles was that it would provide parents with intensive, interactive, fun, culturally sensitive, non-judgmental opportunities to shape their home environments and lifestyle practices to protect their children’s health. Furthermore, it would support parent-child interaction and child development, help parents develop realistic, effective plans that empower their families, and promote positive strategies and changes that parents can control in their home environments to reduce the risk of excessive weight gain in their preschoolers [15,83,124]. A positive approach teaches parents what they can do (eat more fruits) rather than giving them prohibitions (cut out fries) [125,126]. Considerable evidence demonstrates the value of promoting positive rather than restrictive behaviors to achieve health goals—imposing dietary restrictions can lead to increased preference for prohibited foods and promote a return to previous eating habits when restrictions are removed [19,127].

### 2.4. Program Structure

The HomeStyles intervention instructional materials are comprised of 12 brief (~15 min each) guides (*i.e.*, lessons), with each guide focusing on strategies parents can use to shape one aspect of the home environment and lifestyle (see Table 1 and next section below). With the exception of the first guide (Healthy HomeStyles), which provides orientation information for using the other guides, all guides are independent from each other to enable parents to control the sequence they choose the guides and omit guides that focus on an aspect that does not affect their family (e.g., if families watched little or no television, they may not need the Taming TV guide).

**Table 1:** HomeStyles guide content: Description and main concepts.

Description and Main Concepts
**Healthy HomeStylesThis**
This Guide sets the stage for participating in HomeStyles. All families complete this Guide first. Eating, playing, and sleeping choices affect the health of the whole family and can lead to lifelong habits. HomeStyles helps families make simple changes to stay healthy that can lead to big improvements. Kids copy their parents—so it is important to be a good role model and take responsibility for family decisions. HomeStyles helps parents set and reach small, easy goals that help make changes towards a healthier future.
**Family Mealtimes**
This Guide gives parents the secrets to successful family meals. Families do better when they eat together. Kids are happier and feel good about themselves. Kids feel more secure and closer to their families. Kids do better in school. Mealtime chats help kids learn how to say new words, make sentences, and listen. Older kids are less likely to drink alcohol, smoke, or use drugs. Families who share meals get health benefits, too. Their meals are healthier. Healthy meals mean a healthier family! Kids are less likely to be overweight.
**Enjoyable Mealtimes**
This Guide helps parents have calmer, more relaxed family meals. Sharing time together at meals strengthens families. Mealtime chats promote kids’ brain development. Meals are a great time to catch up and keep in touch with kids’ activities. Calm, relaxing mealtimes help prevent unhealthy eating behaviors. A cheerful mood at meals is linked to eating healthier foods. Calm family meals make it easier for kids to try new foods and learn to enjoy them.
**Right Sizing Portions**
This Guide helps parents serve food portions that are “just right”—and keep body weights healthy. Many people do not know that they are eating portions that are too big, which can lead to overeating and weight gain. Healthy portion sizes help kids grow normally. Healthy portions give kids and parents the nutrients they need. Kids and parents get the right amount of calories to keep weights healthy.
**Fuss Free Feeding**
This Guide helps parents teach kids to enjoy new, healthy foods without fussing. The whole family wins when parents use positive feeding practices. Kids have fewer mealtime tantrums. Kids learn to enjoy eating healthy foods. Kids eat more healthy foods, like fruits and vegetables. Kids have healthier weights.
**Taming TV**
This Guide helps parents swap TV-time for active playtime and reduce the effects of TV on kids. People who watch TV more than 2 h a day may have problems. Children may have trouble learning and not do well in school. Kids may have problems getting along with others, especially if they watch television programs made for adults. Many kids and parents who spend too much time watching TV have health problems, like diabetes and heart disease. Many also have excess body fat and eat less healthy meals and snacks. People may overeat when they eat while watching television because they pay attention to TV, not how much they eat. Individuals learn unhealthy food practices from television advertisements and from seeing favorite characters eat sugary, fatty foods.
**Breakfast, the Right Start**
This Guide helps get the whole family off on the right foot every day. Breakfast helps kids do better in school. Eating breakfast improves memory. Breakfast gives kids energy to run, play, learn, and grow. Breakfast eaters get more of the nutrients needed for good health. They have healthier levels of cholesterol in their blood. Breakfast eaters have healthier body weights. Breakfast skippers get so hungry they are likely to overeat unhealthy foods later in the day.
**Best Drinks for Families**
This Guide helps families go for tasty, guilt-free beverages. Having a sugary drink once in a while is fine. Many people drink more than is healthy. Having sugary drinks every day can cause problems for parents and kids. They get too few vitamins and minerals. They get too much sugar. They get more calories than they need. Having sugary drinks often may lead to weak bones, cavities, and too much body fat.
**Play More, Sit Less**
This Guide helps parents trim screen-time and get more family fun time. Getting more than 2 h of screen-time each day can cause problems. Too much screen-time can harm kids. They may have shorter attention spans and learning problems. Many have poorer reading skills. Children may misbehave more and have poorer social skills. Most sleep poorly and feel tired. Kids eat less healthy meals and snacks. Boys and girls gain excess body fat, which can lead to severe health problems.
**Time to Play**
This Guide helps families play more and have lots more fun together. Many kids and adults do not spend enough time in physical activity—they should get 60 min each day. Families get benefits like these when they are physically active. Playing together as a family promotes closer family bonds. Families who play together feel better about themselves, sleep better, have lower stress levels, fight off illness more easily, have fewer health problems, have stronger bones and muscles, have healthier blood pressures, and have healthier body weights.
**Good night, Sleep right**
This Guide helps families get enough sleep and wake up happy and rested. Many kids and adults do not get enough sleep. Kids who do not get enough sleep may have many problems. They have a harder time learning and remembering. Kids are more likely to fall and get hurt. Many are short-tempered and misbehave. Children may have excess body fat.

### 2.5. Delivery Mode

HomeStyles Guides were designed to be delivered electronically (website in English and a mirror site in Spanish, by email, and/or eBook) or face-to-face by home visitation staff during regular home visits. An electronic delivery mode was developed because 80% of the U.S. population uses the Internet (up from 9% in 1995) [128], and according to the Federal Communications Commission, 87% of U.S. families with minor children have computer access at home [129]. The American Academy of Pediatrics supports home-based parent education programs [130] and research indicates that home-based programs hold promise for childhood obesity prevention [131], yet few materials focusing on key obesity prevention strategies are available. Varied delivery modes were used because parents need information source options that fit their lifestyles, learning styles, and desired format [128,129,130,131,132,133].

### 2.6. Guide Content

An extensive literature review was conducted to determine the most salient factors affecting childhood obesity to address in the home environment [116]. To further ensure that the guides addressed the most important topics, the advisory group was consulted. Three key areas emerged: diet, physical activity, and sleep. The vast majority of children spend time in childcare [134]; thus, a common thread woven throughout the guides was to help parents develop skills needed to advocate to their childcare providers for settings supportive of healthy weights. The research supporting the selection of topics addressed in the HomeStyles guides is summarized below.

**Diet**. Despite having the most abundant food supply ever, national data indicate that most Americans eat fewer servings of fruits and vegetables than recommended and few meet dietary intake recommendations [135,136,137,138]. In addition, fat and sugar intake for all age groups is higher than considered supportive of good health and has been linked to increased risk for obesity [139,140,141,142,143,144]. Nutrition education interventions can improve dietary intake and alter food selections [145,146,147]. Reshaping the aspects of dietary intake described below could make an important contribution to reducing obesity risk.

***Encourage More Fruit and Veggie Availability and Intake***. [46] Americans eat too few fruit and vegetable servings [148,149]. Children and teens from limited resource families are least likely to eat these foods [150,151,152]. Diets consistently rich in fruits and vegetables are linked with reduced risk for many chronic diseases, including obesity [50,125,139,153]. Increased intakes of higher fiber forms of fruits and vegetables may be an effective weight control strategy [50,53,125,154,155].

***Rethink Beverage Choices (i.e., Reduce Sugar-Sweetened Beverage Intake and Increase Water and Reduced-fat Milk Intake)***. Soft drink consumption has increased dramatically and is a risk factor for obesity [156,157,158,159]. By age 5, soft drink intake exceeds that of 100% fruit juice and by age 13 soft drinks exceed intake of milk, 100% fruit juice, and fruit drinks [160]. Caloric sweeteners in beverages add ~175 calories/day to the diets of Americans aged 2 years and older [161,162]. Decreased milk intake and increased sweetened beverages likely are contributing to the obesity epidemic [58,139,158,163,164,165,166,167].

***Encourage Cereal for Breakfast***. Eating breakfast is associated with healthier body weights and better nutrient intakes [168,169,170,171,172,173,174,175,176,177,178,179,180]. Ready-to-eat cereal, even presweetened cereal, is one of the lowest calorie, highest fiber, and most nutrient dense breakfast choices; eating cereal for breakfast is associated with healthier body weights and children’s nutrient intake [168,175,176,179,180,181,182].

***Serve Age-Appropriate Portion Sizes***. Portion sizes of foods served in restaurants, individually packaged foods, and home-prepared foods have increased in tandem with the increased incidence of obesity [6,183,184,185,186]. The portion size of food served correlates positively with body weight and directly influences the amount eaten [187,188,189,190,191,192,193]. That is, people tend to eat greater amounts when served larger portions—without reducing energy consumed at that meal or compensating at other meals [191]. The effect of large portions on intake is particularly worrisome given that one-third of adults report that they base amount eaten on quantity served [188]. In addition, most cannot accurately estimate portion sizes and do not know recommended intake amounts [194,195]. Consumers also do not recognize portions that are larger than recommended or the effect of portion size on body weight [188,196]. Increases in prevalence of overweight may be influenced by a shift in eating patterns towards larger portion sizes of energy-dense foods [62].

***Eat Together as a Family Often***. Family meals appear to be particularly important in helping youth develop healthy eating patterns [26,27,28,29,30,31,32,33,34,197,198,199]. All too often, however, families do not eat together for many reasons including hectic schedules, dual working parents, and lack of interest or ability to prepare meals or increase the quality of pre-prepared meals [200,201,202,203]. When children do not eat family dinners, they are more likely to eat meals of lower nutritional quality [203,204,205,206]. The frequency of family meals with at least 1 parent present is positively correlated with intake of fruits, vegetables, calcium-rich foods, grains, fiber, folate, iron, and vitamins B6, B12, C, and E and negatively correlated with intake of sugar-sweetened soft drinks and saturated fat [203,204,205,206]. Family meals have the potential to help children avoid unhealthy weight gain because the nutritional benefits associated with family meals are linked with successful weight control.

***Promote Positive Family Mealtimes***. Family meals also contribute to children’s positive psychosocial development [197,207]. However, the content of family mealtime conversations may determine whether psychosocial and health outcomes are positive. For example, research regarding family mealtime recollections revealed that, relative to normal or underweight young women, overweight young women recall family discussions that may have contributed to their current weight status [208].

Mealtime distractions such as TV can adversely affect dietary quality [29,206,209,210]. TV is on during dinnertime four or more times each week in more than one-third of American households [206]. TV was on during 238 of the 240 home visits to observe maternal feedings of infants in low-income families [211]. Television use during mealtime may reduce family interactions and is associated with poorer eating choices [29,206,209,210,212]. Families who routinely watch TV during mealtimes include fewer fruits and vegetables and more pizzas, snack foods, and soft drinks than families who separate eating and TV viewing activities [213,214]. Avoiding TV watching at mealtime may support the development of obesity-preventing eating patterns.

***Promote Positive Parental Feeding Practices***. Within the context of the family home environment, especially during mealtime, children learn important values and lessons about eating [6,36]. These lessons may be taught by instruction, modeling, reinforcement, and exposure to foods [206]. Lessons with the most positive outcomes appear to be those providing children with multiple opportunities to become familiar with new foods and modeling healthful eating behaviors by parents [6,23,24]. For example, children’s intake of and preference for nutrient-dense foods are linked to availability in the home, parental intake, and parental willingness to let children decide how much to eat [16,17,18,19,20]. In contrast, pressuring a child to eat a particular food or offering a reward to finish a food decreases desire for the food and subsequent intake [215,216,217]. Additionally, restricting access to palatable foods, such as sweets, can elevate the attractiveness of these foods and compel children to eat these foods even when they are not hungry [16,19,218]. Controlling parental actions or “food rules” also may precipitate other undesirable outcomes, such as impairing children’s eating self-control and ability to self-regulate food intake, and may be linked with behaviors like binge eating and dieting in adulthood [19,218,219,220,221,222,223,224]. Positive parental feeding practices help children develop eating regulation skills that will help them maintain a healthy weight [6].

***Tame the Effects of TV on Diet***. The American Academy of Pediatrics recommends limiting preschoolers’ screen-time (watching TV, playing video games, using computers) to two hours/day [225]. Nearly one-third of preschoolers surpass these guidelines just by watching TV. Children exceeding these guidelines are more likely to be overweight than those who limit screen-time [48,226]. Excess screen-time affects diet (*i.e.*, more snacking while watching TV and seeing more TV ads promoting calorie-rich foods) and physical activity (*i.e.*, substituting screen-time for exercise) [31,159,226,227,228,229]. TV ads increase children’s food requests and frequency of snack and fast food intake and thus affect diet and BMI [64,65,66,68,209,230,231,232,233,234,235,236]. Substituting other activities for screen-time can help children maintain healthy weights.

***Physical Activity***. The most easily influenced aspect of energy expenditure is physical activity [237,238,239]. People of all ages benefit physically and mentally from regular physical activity [240,241,242]. Despite numerous potential benefits, more than 60 percent of American adults and about half of youth are not vigorously active on a regular basis [242]. Because physical activity and fitness both track over the lifespan, it is important for children to establish positive lifestyle habits and healthy levels of fitness [2,3]. The aspects of physical activity addressed in HomeStyles (e.g., play together often as a family, substitute active play for sedentary activity) were selected because they tend to be problematic and by reshaping them, families could help children to get active to maintain healthy weights.

***Set Aside Time for Fun, Active Family Playtime***. The influence of parental modeling of physical activity along with their beliefs about physical activity and encouragement they provide to children are important predictors of children’s physical activity levels [7,8,243]. Identifying enjoyable, affordable, age-appropriate activities that parents and children can do together regardless of weather conditions is important for establishing lifelong exercise patterns [71,244].

***Trade Screen-time for Active Play***. Substituting active play (either physical exercise or active screen-time such as exercise or dancing videos, active video games) for sedentary screen-time can help families keep body weight under control [225,244,245,246,247].

**Sleep**. Sleep duration also may influence the development of overweight in preschool children [248]. For example, daily sleep duration of less than 12 h during infancy appears to be a risk factor for overweight and adiposity in preschool-aged children [54]. Preschoolers who get adequate sleep are less likely to be obese [48].

**Childcare**. Nearly 80 percent of children under 5 years spend at least some time in non-parental childcare settings [249]. Preschoolers cared for at home by their parents are less likely to be obese than children cared for by other family members, friends, or neighbors, suggesting a need for a larger parental role in the management of their children when they are away from home [74,75,76,250]. Thus, raising parents’ awareness of the need to advocate for childcare settings that promote healthy weights through supplying healthy meals and snacks, promoting physical activity and limited screen-time, and allowing sufficient naptime could help protect the health of young children.

### 2.7. Guide Development Process

The multi-step development process began with a literature review to establish an in-depth understanding of each guide’s topic with a particular emphasis on the topic vis-à-vis families with preschool children (see these review articles as an example of the literature reviews [197,248]). Additionally, to permit application of Adult Learning Theory principles, focus groups (*n* = 139 parents of preschool children) were conducted in two geographic locations to explore parents’ cognitions, barriers, supports, and modeling of behaviors associated with each topic [198].

In the next step of this process, the “Research & Development” (R & D) draft of each guide was written by a team of nutrition communication experts (Note 1) [251]. This draft was fully referenced and included annotations indicating where Social Cognitive Theory constructs, Faith’s Core Behavior Change Strategies to Treat Childhood Obesity, and motivational interviewing principles were used (see Application of Behavior Change and Motivational Interviewing Strategies section below).

After numerous reviews, reorganization, and refinements by the writing team, the R & D draft was reviewed by experts in the guide’s subject matter (e.g., sleep, family meals, physical activity), as well as experts in health behavior change, motivational interviewing, cultural appropriateness for diverse audiences, health communications and literacy, parenting, child development, and home visitation programs. After extensive iterative reviews and refinements, the R & D guide was formatted into a consumer-friendly document by deleting references and annotations to create a Content Cognitive Testing draft.

The Content Cognitive Testing draft was a text document (12 point type, single spaced, 1 inch margins with bold headers demarcating each section of the guide (Table 2)). Cognitive testing began as soon as the Content Cognitive Testing draft was created; thus guides created later in the development process were refined to incorporate findings from those that were cognitive tested earlier in the process. Home Visitation staff (~3 per guide) and parents (~4 in each of 2 geographic regions per guide) participated in content cognitive tests. Cognitive testing interviews with Home Visitation staff investigated their impressions of the content, its usefulness, the likelihood it would capture the attention of the parents in their home visitation caseload, potential for benefitting parents, needed improvements, and the confidence in their abilities to introduce the guide’s content during home visits.

**Table 2:** HomeStyles guide components *.

**Here Is What the Experts Say**
All Guides start with a brief summary of evidence-based research that explains why the Guide’s topic is important to health.
**Kids Copy Their Parents**
This section helps parents remember they are their children’s most important role model.
**Take a Minute**
These sections give parents a chance to think about why the behaviors discussed in the Guide are important to them personally. These sections also provide opportunities to use motivational interviewing techniques to help families make simple changes to build healthier families.
**Here’s What Other Parents are Saying**
This section provides tips and ideas from actual families with preschoolers. It helps parents know they are not alone, and that other families have successfully made changes to improve their kids’ health.
**Even More**
This section provides more tips and ideas specific to the Guide to help parents raise happier, healthier, safer kids.
**Goal Setting**
This section helps parents set small, attainable goals to improve their kids’ health. Parents can set their own goal or choose from the examples other families have set.
**Remember**
This section sums up the Guide. It also reminds families to take small, manageable steps and remember that the changes they are making are important for their family!

*: All Guides except the Healthy HomeStyles Guide (the first one parent use) have the same parts. The Best Drinks for Families Guide is an example that shows all the parts.

The purpose of the Content Cognitive Testing interviews with parents was to ensure guide comprehension, applicability, and acceptability. During the cognitive testing, parents were instructed to read a section aloud and then asked a series of questions to determine their overall understanding and impressions of the information in the section (what they liked, disliked, and would change) and the degree to which they felt the information would help parents like them engage in the recommended behaviors (e.g., eat family meals more often). After reading the entire guide, parents were asked whether they felt the guide would capture the attention of parents, would be useful to their families, and what improvements were needed. They also rated the guide’s clarity, appeal, relevance, usefulness, and how interesting and motivating they felt the guide was, and how likely it would affect their practice of the recommended behaviors.

The guides were refined based on the Content Cognitive Testing results. A final literacy check was conducted to ensure readability was between fourth and sixth grade, then the content was professionally copy edited and sent for graphic design. The graphic designer, who was a member of the HomeStyles advisory group and thus, fully informed about the details of the project, created an array of Design Cognitive Testing drafts. The designs were 4-page mini-magazines (11 × 17 inches folded) featuring a full page color photograph of parents and/or young children on the cover. The inside and back pages contained written content, photographs, and colorful shading. The same design elements were used in all guides to create a unified, HomeStyles branded look and feel.

**Figure 1:**
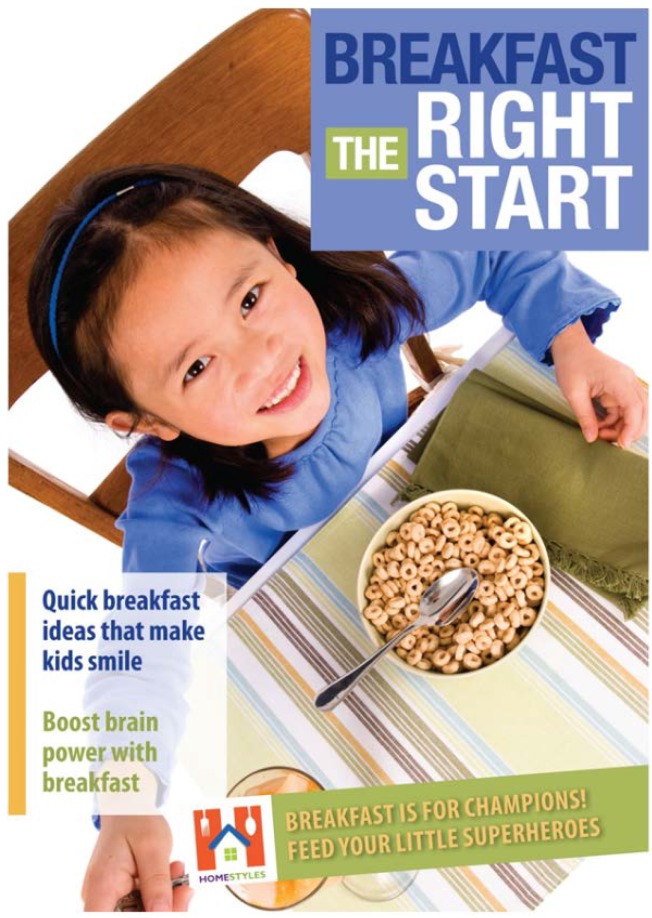
Example guide showing cover lines.

The designs were reviewed by the writing team, refined, and then subjected to Design Cognitive Testing. In addition, the cover design presented the opportunity to incorporate cover lines on the cover page. Cover lines (sometimes called headlines) are short phrases on magazine covers designed to stimulate reader interest [252,253,254,255]. The writing team created cover lines for each guide and evaluated the extent to which mothers of young children (n = 77) felt each cover line motivated them to read a short magazine article [256]. The cover lines rated as most motivating were added to the guide covers (Figure 1).

The Design Cognitive Testing interviews were conducted with parents of preschool children (~5 parents in each of 2 geographic regions per guide). These interviews began by asking the parent to spend a few minutes looking over a guide and then verbalize thoughts, feelings, and ideas that came to mind immediately after exposure to the guide using the think-aloud protocol. The purpose of using the cognitive response method was to confirm that parents’ first impressions of the guide were positive and likely to compel them to engage with the guide and to determine parent perceptions related to the theoretical underpinnings and philosophy embedded in the guides [257,258,259]. Next, parents were asked a series of questions to determine their feelings about the graphic elements (*i.e.*, photographs, color scheme, font, spacing, amount of text *vs.* pictures), layout and presentation of each of the guide components, writing style, and length of the guide. Parents also were asked to indicate the usefulness of the guide, improvements needed, and whether the guide would benefit parents like them. As before, this cognitive testing began as soon as a guide was put into design format; thus, guides created later in the development process benefited from findings related to guides that were cognitive tested earlier. Home Visitation staff (~2 per guide) also participated in the Design Cognitive Testing phase to further gauge their perceptions of the guides’ usefulness, benefit, and needed improvements.

In the last development step, the guides were refined to address Design Cognitive Testing findings, sent for final review by cultural sensitivity and subject matter experts, and professionally proof read. After the Content Cognitive Testing refinements were made, the guides were professionally translated into Spanish using in-culture procedures (*i.e.*, the translations were created to be understandable for all versions of Spanish spoken throughout the world). Then, the Spanish translations were subjected to Content Cognitive Testing and Design Cognitive Testing by parents using the same steps described previously. Spanish-speaking home visitation staff also reviewed the Spanish guides.

## 3. Application of Behavior Change and Motivational Interviewing Strategies

**Figure 2:**
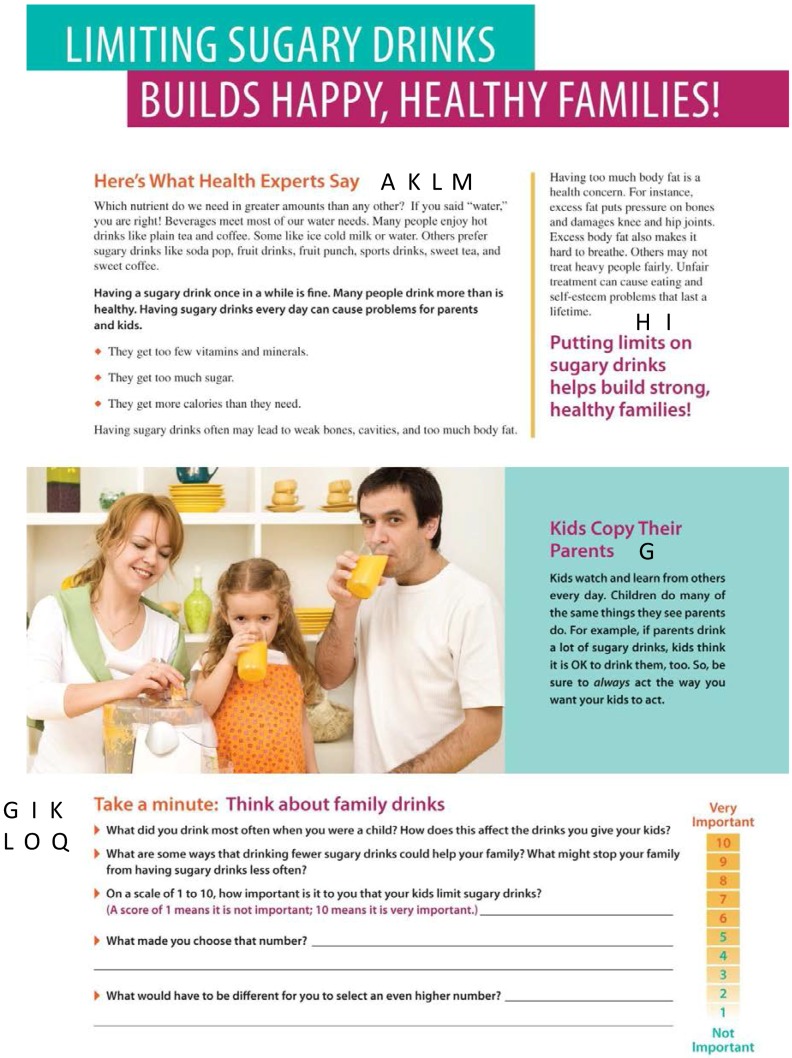

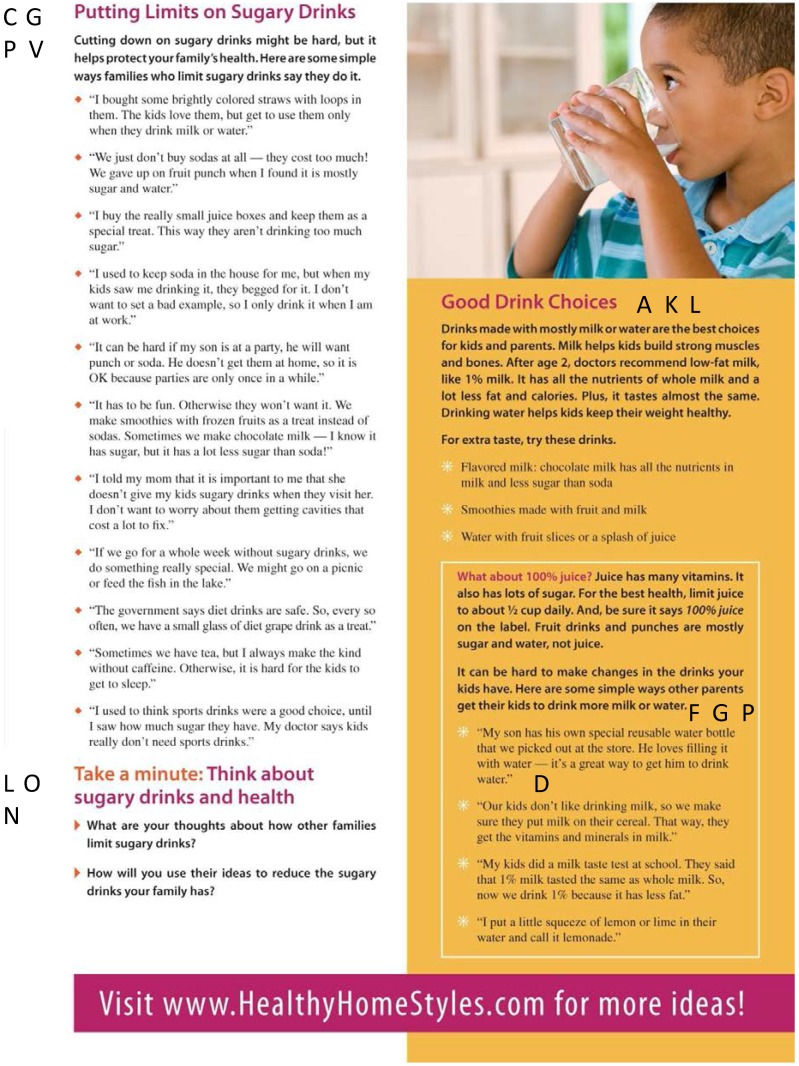

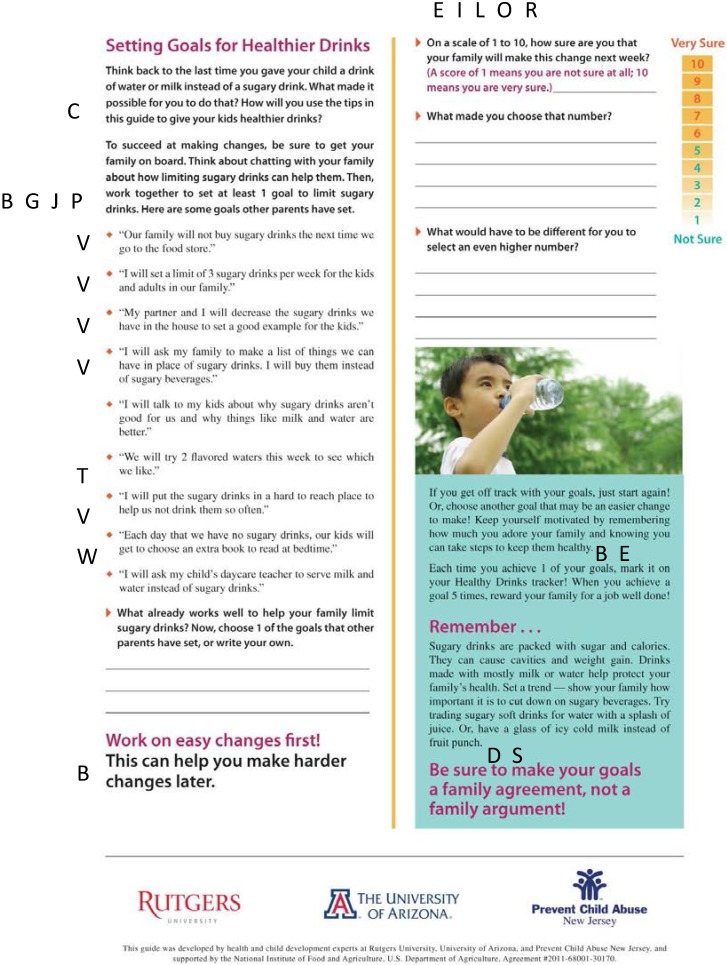
Best Drinks Guide as Example Showing Use of Social Cognitive Theory, Faith’s Core Behavior Change Strategies, and Motivational Interviewing Strategies. Note: The letters in black boxes correspond to the strategies in Table 3.

Table 3 and Figure 2 illustrate where strategies from Social Cognitive Theory (SCT), Faith’s Core Behavior Change Strategies to Treat Childhood Obesity, and motivational interviewing (MI) were incorporated in the body of one HomeStyles guide. All other HomeStyles guides use strategies similarly. Examples from a variety of Homestyles guides are given below.

**Table 3:** Behavior Change and Motivational Interviewing Strategies Used in HomeStyles Guides. Letters in the left column correspond to the letters in black boxes in Figure 2.*

Letters in Black Boxes in Figure 2	Behavior Change Strategy
A	**Outcome Expectations** [91] Beliefs about the likelihood and value placed on the consequences of behavioral choices.
B	**Behavioral Capability** [91] Having the necessary knowledge and skills to change a behavior.
C	**Self-efficacy** [90,91] Confidence in one’s ability to perform a behavior.
	**Supporting Self-efficacy** [2] Giving a person the opportunity to express self-confidence.
D	**Reinforcement** [91] Outcomes that give support (or take away support) for performing a behavior. Most commonly positive reinforcement to reward an individual for making a behavior change.
E	**Self-regulation** [91] Controlling oneself through self-monitoring, goal-setting, feedback, self-reward, self-instruction, and enlistment of social support
F	**Address Barriers** [91,106] Identify real or perceived factors preventing behavior change. Also called Roadblocks.
G	**Observational Learning/Modeling** [91] Learning to perform new behaviors by exposure to interpersonal or media displays of them, particularly through peer modeling
H	**Eliciting Change** [106] Examines reasons for changing a behavior.
I	**Exploring Importance** [106] Examines importance of changing a behavior.
J	**Goal-Setting** [90,91,106] Setting goals for changing a behavior (related to Self-regulation).
K	**Rewards** [106] Identifies benefits of changing a behavior that are most important to a person.
L	**Relevance** [90,106] Examines why changing behavior is important to a person.
M	**Risk** [106] Identifies the risks that a person feels are most important to avoid.
N	Repetition [106] Revisits questions when a person indicates resistance/ambivalence to changing a behavior.
O	**Reflection** [106] Asking open-ended questions that give a person an opportunity to think and reflect.
P	**Normalizing** [106] Helping a person to understand that personal feelings/experiences/challenges while making change are common and normal.
Q	**Decisional Balance** [106] Comparing “good” and “not so good” outcomes about changing a behavior.
R	**Readiness to Change Scale** [106] Rating change reading using a 10-point scale where 1 = definitely not ready to change and 10 = definitely ready to change.
S	**Summaries** [90] Reminds a person of main aspects of changing behavior made in the current session.
T	**Specifying Target Behaviors** [90] Identifying the *specific* behaviors that need to change.
U	**Self-Monitoring** [91,106] Keeping track of specific behavior that is targeted for change each time it occurs.
V	**Stimulus Control** [90] Changing and structuring the environment (usually the home) to make it easier to perform a behavior (e.g., eat healthier foods) or avoid performing a behavior (e.g., eating unhealthy foods).
W	**Positive Reinforcement Strategies** [90,106] Using praise and recognition of changes that have already occurred to encourage change.

*: All strategies used in HomeStyles guides are described in this Table. All guides used the vast majority of the strategies, but not all were used in every guide.

The Here’s What the Experts Say section of the guides addresses the SCT construct of outcome expectations (e.g., “The HomeStyles program helps families make simple lifestyle changes to stay healthy…small changes can add up to big improvements.”, “Families do better when they eat together.”, and “Pleasant family meals are an easy way to have fun, feel closer and eat healthier.”), which, in turn, helps elicit change (MI). Additionally, SCT and MI strategies of identifying barriers or roadblocks to behavior change (e.g., parents may feel too tired to play with kids) and Faith’s and MI strategy of relevance (e.g., “choices affect the health of the whole family”) were incorporated in this section. This section also introduces risks associated with unhealthy behaviors (MI; e.g., “Some people may treat heavy kids unfairly or tease them. Teasing can cause lifelong eating, weight, and self-esteem problems.”).

The Kids Copy Their Parents section acknowledges that children learn through observation (SCT; e.g., “*Kids do many of the same things they see their parents doing*.”). This section also reinforces (SCT) to parents that they are important role models for their children.

The Take A Minute sections of the guides used MI concepts of reflection and relevance by incorporating open-ended questions (e.g., “*How often did you eat family meals when you were a child? How does this affect what you do now?*” “*What are some ways that active playtime could help your family?*”). These questions provide an opportunity for parents to think about how they feel about a behavior (attitudes) and explore the importance (MI) a behavior change is to them. A readiness to change scale strategy from MI was incorporated to assess the importance parents placed on adopting a behavior and prompted decisional balance (MI) activities of comparing possible outcomes (SCT; e.g., “*On a scale of 1 to 10*, *how important is it to you that most of your family eats together as often as they can? What made you choose that number? What would have to be different for you to select an even higher number?*”) This section also incorporates Faith’s and SCT construct of self-efficacy (e.g., parents think about what they already do to keep their family healthy which can build confidence).

Similarly, the Here’s What Other Parents Are Saying section of each guide incorporated advice from peers to help parents normalize their own experiences and challenges in making behavior change and help them reflect on how they might try similar strategies (MI strategies). Providing real-life examples helps increase self-efficacy (Faith’s and SCT construct) and supports the SCT constructs of observational learning and behavioral capability by providing knowledge and information about the skills other families used to perform a behavior (*i.e.*, peers of the parents: “*Here’s how families who use positive feeding practices say they do it*…”).

The Even More section provides another opportunity to reinforce the SCT constructs outcome expectations (“*Milk helps kids build strong muscles and bones*”), observational learning (through tips from other parents), and behavioral capability (“*Serve meals and snacks in child-size bowls and plates*”). The title for the Even More section varied for each guide (e.g., Putting Limits on Sugary Drinks, Eating Healthy Portion Sizes).

The Goal Setting section of each guide encourages parents to set small, attainable goals to improve family health. Examples of goals other families had set (e.g., “*I will start using measuring cups and spoons to learn what healthy size portions look like.*”; SCT concept of observational learning) are included, and parents are encouraged to select one of these goals or set their own goal (SCT, MI, and Faith strategy). MI goal-setting strategies of making goals specific, measurable, assignable, realistic, and time-related were used. Behavioral capability (SCT) was encouraged (“*Work on easy changes first! This can help you make harder changes later!*”). A final readiness to change scale (MI strategy) was included to assist parents with creating attainable goals. The goal tracker sheet, which accompanied guides, included ideas for non-food, no-cost rewards that parents could use with their families to provide positive reinforcement for making changes (SCT).

The Goal Setting section of the guides also incorporated Faith’s strategies by asking parents to specify the target behavior to change and to use a tracking sheet for self-regulation, self-monitoring (SCT and MI) of the behavior. This section also included tips for changing and structuring the home environment to support behavior change through stimulus control and with positive reinforcement strategies or rewards (e.g., “*After my daughter eats a fruit or vegetable*, *we put a sticker on her belly*.” Faith’s and MI strategy).

The Remember section of the guides provided a brief summary of the primary topic addressed in the guide and repeated the core messages (MI). Self-regulation was also reinforced through enlisting social support (SCT) (“*Be sure to make your goals a family agreement*, *not a family argument*”).

## 4. Cognitive Testing Findings

A total of 39 Home Visitors and 92 English-speaking and 120 Spanish-speaking parents participated in the Content Cognitive Testing. For the Design Cognitive Testing, 120 English-speaking and 121 Spanish-speaking parents as well as 20 home visitation staff members participated. Parent interviews were distributed approximately equally between New Jersey and Arizona. Home Visitors were recruited through their workplace (state, county, and local community agencies). Parents were recruited through community centers, workplaces, and religious groups. All participants gave informed consent.

### 4.1. Home Visitor Content Cognitive Testing

The guides were well-received by the Home Visitors who indicated the guides were “*important*” and “*helpful*”. They felt the guides were “*concise and clear*” and indicated that the tips and examples from other parents included in the guides were useful (“*gives another point of view*”) and the focus on goal-setting was a particularly effective strategy (“*Asking families to set goals makes them have the power to change and makes them more likely to stick to the goal*.”). Suggestions for improvement included making the wording clearer and balancing written text with graphics to avoid overwhelming parents. A few Home Visitors expressed safety concerns about involving children in food preparation and felt more attention should be given to safety in the family mealtime guide. They also anticipated challenges in presenting the positive/calm meals guide: “*Families might not think there is a problem to fix…show examples of what a calm family mealtime is like and what a chaotic one is like*”. Because “…*a lot of parents think that the TV is a good babysitter*,” a few Home Visitors felt parents might resist the idea of reducing screen-time.

### 4.2. Parent Content Cognitive Testing

Overall, parents could read and understand the content and felt it was “very useful”, “informative about things I should know”, “reading this gives me the idea to actually play with them instead of just sitting there and watching them”, “most parents don’t know that cereal can be healthy”, and “helpful in getting parents to think about how to make changes”. Parents also enjoyed the guides (“I liked it because it gives fun ideas [to help families make changes]”) and found them “attention-capturing”, “logical”, and “easy to understand”. General suggestions for improvement included considering local variations in food availability and seasonality, addressing perceptions of what healthy foods are, and identifying areas to tighten to reduce repetition. Misconceptions that warranted greater clarity and/or emphasis included the healthfulness of canned and frozen fruits and vegetables, portion size (“I don’t pay attention to portion sizes. It makes me feel good to see people eating.”), and feeding practices (“I do not agree with forcing your child to eat. (But) sometimes, we as parents do it…because we have very little food.”). Both English and Spanish speakers suggested adding recipes (“it would be nice to have 1 or 2…”).

Table 4 describes the parents’ ratings of the guides’ content. Using a scale of 1 (low) to 5 (high), parents agreed to strongly agree that the guides were interesting, useful, relevant, clear, easy to read, appealing and likely to improve their practices related to the guide content. They also felt the amount of time needed to read the guides was just right and the guides’ tone was positive. After reading the guides, parents felt their knowledge increased and that they would place more importance on engaging in the practices described in the guide.

**Table 4:** Cognitive testing content participant rating of HomeStyles guides.

Guide Content Characteristic	Mean ± SD
Interest Level	4.70 ± 0.68 ^a^
Usefulness	4.78 ± 0.59 ^a^
Relevancy	4.62 ± 0.72 ^a^
Clarity	4.66 ± 0.63 ^a^
Reading Ease	4.65 ± 0.65 ^a^
Appeal	4.50 ± 0.93 ^a^
Likelihood to Improve Practices	4.53 ± 0.82 ^a^
Time Needed to Read	2.09 ± 0.41 ^b^
Tone	2.91 ± 0.30 ^c^
Knowledge Change after Reading Guide	2.88 ± 0.32 ^d^
Importance Placed on Engaging in Practices Described After Reading Guide	2.90 ± 0.30 ^d^

^a^: 5-Point scale; 1 = strongly disagree, 2 = disagree, 3 = neither agree nor disagree, 4 = agree, 5 = strongly agree; ^b^: 3-Point scale; 1 = too short, 2 = just right, 3 = too long; ^c^: 3-Point scale; 1 = negative, 2 = neutral, 3 = positive; ^d^: 4-Point scale; 1 = poor, 2 = fair, 3 = good, 4 = outstanding; ^e^: 3-Point scale 1 = less, 2 = same, 3 = more.

With regard to specific sections in the guides, parents asked for greater clarity on the source of the information in the evidence-based research (Here’s What the Experts Say). The section that reinforced the importance of role modeling (Kids Copy Their Parents) was well received (“*I enjoy how it discusses setting the right example for the kids without criticizing the parents.*”). They also agreed that children copy parental behaviors: “*It’s right on. My kids copy my husband’s taste in food. If he doesn’t like it*, *they won’t eat it either*”. Parents appreciated the inclusion of tips and ideas from other parents who had successfully adopted healthy behaviors (Here’s What Other Parents are Saying and Even More sections). Parents indicated that these sections “*give another point of view*”, “*It’s good that* (the tips) *are coming from other parents*; *parents don’t always take ‘professionals’ seriously because they don’t know if the person giving them advice has ever had the experience with the child*…” and “*I can see other people like me who have a hard time getting the family together for dinner* (we’re able to have family meals)”. In particular, both English- and Spanish-speaking parents appreciated healthy breakfast tips (“*It’s a good idea*, *putting the* (cereal) *bowls out the night before*”) and ideas of indoor activities (“*They give us ideas with different activities*, *not just the typical go-to-the-park example*.”). However, the perceived usefulness of certain tips varied. For example, in the family meals cognitive interviews, some Spanish speaking parents disagreed with the guide’s tips for keeping meals calm by engaging children in conversation by telling jokes (“*We are at the table to eat*, *not to have fun*”) and found the phrase “fast meals” confusing (“*It should not be a quick process eating as a family*”).

Most parents indicated that the reflection questions, which use motivational interviewing strategies (Take A Minute sections), were “*helpful*” and “*got them thinking*”. Similarly, most parents felt the Goal Setting section was beneficial (“*Goals that other parents have done give me a starting point*.”) and appreciated the accepting tone of the Remember section, which reminds parents to take small, manageable steps and to just “start over” if they “get off track” (“*I like the ‘remember’ part which says to love your family.*”). However, a few English-speaking parents felt the Goal Setting section was demanding (“*It sounds like people are being forced to set goals*; *nobody can be forced*” and “*I don’t need to set a goal to eat. I like having a pleasant meal but not to make it a job*.”). A few felt goal-setting would take too much time (“*I don’t have time to write everything down*…*it would be useful…but needs to be simplified*.”).

### 4.3. Home Visitor Design Cognitive Testing

The Home Visitors liked the design of the guides overall. They had positive comments about the pictures, colors, and design features. Some mentioned how the design features and color combinations were “eye catching” and helped emphasize information (“*like how certain features were highlighted*”) and that the pictures included families from different cultures (“*I like the pictures*, *different cultures*, *no focus on one particular* (culture)”). They liked that healthy foods were promoted in guides (“*good*, *they are encouraging fruits and vegetables*, *water*, *no soda*”) and that pictures included “*parents…actually interacting with the kids*”. Some also requested the use of more pictures.

Home Visitors found the guides to be “*informational*, *useful*, *and believable*” and thought they would be helpful for their work with families as “*families ask me for nutritional guidance*”. The guides were seen as promoting positive behaviors as many Home Visitors often used the word “*happy*” when describing their initial impressions of the guide. They thought that the Here’s What the Experts Say section had good information and was “*a good reminder even if you know about eating healthy*”. The Kids Copy Parents section was very popular. They felt the Take A Minute Section provided a time for parents to reflect and do some “*self-evaluation…do I like where this is going? Or should I make changes?*”. Most thought that parents would relate to and enjoy the examples of positive behaviors and changes other families have made. The Goal Setting techniques used in the guides were well received by the Home Visitors as they use similar strategies in their current work with parents, and felt that it was a good reinforcement. Several Home Visitors suggested creating video versions of the guides to make them more useful to their clients and having it available in languages other than English and Spanish.

### 4.4. Parent Design Cognitive Testing

This phase of cognitive testing indicated that parents’ first impressions were that the guides were positive (“Looks cozy, fresh. I love the colors”, “Any parent will pick it up”) and engaging (“As soon as you pick it up and start reading, you’re going to want to finish it.”). Parents appreciated pictures of families from many cultures (“Nice diversity because we all have to eat the same no matter what color we are.”), liked the bold, eye-catching colors, and appreciated the balance between the text with graphics (“The photos help a lot. They show what should be eaten.”). Parents suggested enlarging the font size. They felt the guide length was acceptable (“I’m not a reader, but it’s just right.”) and appreciated the positive writing style (“It like it. (It’s) ‘guilt free’. I would read it.”).

Parents felt the guides were useful, although some gave mixed feedback about the usefulness of the rating scale in the Take A Minute section asking them to indicate how important achieving a particular goal would be to them personally, but conceded it “*may be useful to others*”. Several parents suggested changing the scale colors (e.g., “*Red is like the color of an alert*, *so it should be on the bottom instead of the top*.”). Some found the tables showing portion sizes (in Right Sizing Portions and Fabulous Fruits and Vegetables guides) difficult to interpret (“*It is easier to understand comparisons like the size of a baseball or softball*” and “*Fractions might be confusing for some people…I’ve never given my children fruits and vegetables in cups.*”).

The guides were well received, easily understood, and parents expressed great interest in the content. Parents agreed that the guides would benefit parents like them (“*It has already benefitted me*”, “*It will help me be a role model for my daughter*”, and “*I’m always rushing and going crazy*, *so now I can plan ahead and pack something* (for breakfast)” (Note 2) [260].

## 5. Comparison of Cognitive Testing Findings with HomeStyles Theoretical Underpinnings and Philosophy

A qualitative examination of cognitive testing responses in comparison with constructs of theories and strategies guiding the development of HomeStyles indicated that most parents appreciated the use of motivational interviewing strategies. For instance, comparisons of parent responses to the themes emphasized in motivational interviewing (e.g., non-judgmental, non-confrontational interviewing and empathetic, supportive, and reflective listening to help clients clarify strengths and valued goals and aspirations) showed parents felt the guides incorporated these characteristics. In particular, they felt the guides were non-judgmental and non-confrontational (“*It’s guilt free*”. “*I enjoy how it discusses setting the right example for the kids without criticizing the parents*”. “*It’s good*, *these tips remind you what you should do*”. “*It makes you think about why it’s important and what works or could work for your family*”. “*Even if you can’t do these things right away*, *you can use them to jump off from.*”), empathetic (“*I like the part about starting over if you get off track*”. “*Quotes* (from other parents) *are good. I can see other people like me who have a hard time getting the family together for dinner*”. “*Especially for single moms like me*, *it gives new ideas*”. “*I like these because it sounds like regular people so I can relate*.”), supportive (“*It gives ideas of what to do*”. “*As we go*, *I get more excited and confident.*”), and promoted reflection (“*The questions are helpful. They make it personal by asking what the family does and keeping the emphasis on you*”, “*I think this is helpful in getting parents to think about how to make changes.*”). However, a few were uncertain about reflecting (“*It’s not clear why we’re reflecting on this*”. “*It’s confusing to me. Am I asking questions to myself? Should I just think or write about them too?*”) or felt it was not something they would do (“*It’s alright. You would only answer it if you were going to a dietitian*”, “*I wouldn’t answer it...this reminds me of school*, *like I have homework.*”). Additionally, parents believed the guides helped them clarify their strengths and goals (“*I would like to get my kids excited for meal times and this would help*”, “*This section helps you set an active goal and keeps you aware of the changes you are going to make*”, “*The goals would help my family so we can make changes*”).

Cognitive testing responses also indicated that parents appreciated the use of Adult Learning Theory. Comparison of parents responses to themes emphasized in Adult Learning Theory (e.g., need for relevant examples and problem-solving/goal-oriented instructional approaches) showed most parents felt the guides would facilitate and motivate them to make changes. (“*I can apply these* (ideas) *to our home*”, “*Goals are good…it felt doable*.”). They also indicated the guides contained relevant, applicable, practical, useful information (“*Interesting*, *it helps and is very useful information*”. “*It makes you think about why it is important and what works or could work for your family.*”) that encouraged them to apply it to solve problems and reach goals (“*It is really good*, *it introduced me to keeping my goals every day and I like that*”, “*This goal section can help us come up with different goals my family and I really like*”, “*Examples of goals are good so that people can begin to develop their own ideas.*”). Some, however, noted that reaching goals was hard (“*Many times*, *you do not have the opportunity to provide your children with a variety of foods*”, “*We are often told to do stuff like this but we don’t have time to do it. It can be difficult*, *but it’s good in theory*”, “*It is difficult to practice this in my home because I work late at night.*”).

Cognitive testing results revealed that parent perceptions of the guides were congruent with the philosophical basis for HomeStyles. Parents felt the guides were intensive (“*I think it gives great motivation because the information is already there. Now it is just up to me to implement*”, “*Makes you think there are so many different ways you can change your habits.*”), interactive (“*These are good questions that you need to ask yourself*”, “*It is good because there are many different examples to choose from.*”), fun (“*It brought up fun ideas and helpful advice*”, “*I feel like this is a good idea because it gets the family working together and we can make it a fun activity or routine*”, “*It will help me to get my family closer and to have more fun together.*”), culturally sensitive (“*Pictures are multicultural. Diversity is refreshing*”, *I liked it since it is from other parents with different situations*”, “*I like it because it incorporated my Latino background in the portions section*, *like the beans*, *rice and tortillas*”, “*it represents Hispanics*.”), and non-judgmental (“*I enjoy how it discusses setting the right example for the kids without criticizing the parents.*”). The guides were observed to be supportive of family interaction (“*These are good ideas to help a family start eating together. Those families who are already having family mealtimes can get ideas on how to keep it going*”, “*Focuses on families and keeping them healthy*”, “*Really great section with great examples for children of all ages*”. “*I like the kids participating and showing that everyone needs to work together*”, “*It encourages family time*, *healthy eating*, *and accountability of making good food choices.*”), helped parents develop realistic plans (“*It helps parents to get thinking and discussing what is important*”, “*It makes you think there are many different ways you can change your habits*”, “*Good ideas*, *if you set goals there is a greater chance you will achieve them.*”), and promoted positive strategies (“*I think it is good because it shows positive ways you can apply the info*”. “*I can relate to this! I can picture myself and my daughter doing this*”, “*It is positive to think about trying these ideas.*”). A few, though, wanted less information (“*I am not interested in reading what other families do*, *only the facts.*”) and felt as though “*the guide assumes there is a problem*”.

## 6. Discussion

Ongoing involvement of stakeholders throughout the development phase of interventions is critical to the creation of materials that resonate with them [261]. HomeStyles guides are well accepted by the target audience (parents of preschool children), likely because of their involvement during the design phase and the responsiveness of the research team to parents’ wants and needs. In addition, parents appreciated the positive writing tone employed, recognition of their life experiences, and application of information to reach their goals.

These guides are innovative and novel in several ways, including being among the first nutrition education materials to focus on preventing obesity in children less than age 5 [15,36,78,262,263,264] and using a multifactorial approach to address a broad array of factors associated with increased obesity risk [15,48]. Additionally, they emphasize factors parents can change easily, quickly, and at low or no cost in the home environment—an environment that remains understudied [98,265,266,267,268].

Few studies have investigated parent-led home environment “makeovers” designed to shape environments and lifestyle patterns to be more supportive of optimal child health; however, the results of studies that have been conducted indicate home makeovers hold great promise for attenuating childhood obesity [232,269,270]. Thus, the next phase of the HomeStyles project is to conduct a randomized controlled trial centered on the HomeStyles guides to establish the potential of HomeStyles to be an effective, population-level, sustainable obesity-prevention intervention that enables and motivates parents to shape their home environment and lifestyle practices to prevent excessive weight gain in their preschool children (ages 2 to 5 years).

## 7. Conclusions

Preliminary testing of the HomeStyles guides suggests that the format is well-received and perceived both by parents and Home Visitation staff as an effective tool for helping parents of preschoolers build a healthy home environment. Collaborating with stakeholders throughout the design process and incorporating motivational interviewing techniques and adult learning theory likely helped ensure that the learning experiences and strategies employed will be relevant and effective for the target audience. Further research is underway to test these guides as part of an in-home randomized control trial.
